# Multifocal Avian Influenza (H5N1) Outbreak

**DOI:** 10.3201/eid1310.070558

**Published:** 2007-10

**Authors:** Ran D. Balicer, Shmuel Reznikovich, Elyakum Berman, Michael Pirak, Amnon Inbar, Shimon Pokamunski, Itamar Grotto

**Affiliations:** * Ben-Gurion University of the Negev, Beer-Sheva, Israel; † Israeli Ministry of Health, Jerusalem, Israel; ‡ Veterinary Services of the Israeli Ministry of Agriculture and Rural Development, Beit Dagan, Israel

**Keywords:** Influenza A virus, H5N1 subtype, disease outbreaks, disease transmission, public health practice, dispatch

## Abstract

During March 2006, an outbreak of highly pathogenic avian influenza (H5N1) occurred in multiple poultry farms in Israel. The epidemiologic investigation and review of outbreak mitigation efforts uncovered gaps in planning for and containing the outbreak, thus affording valuable lessons applicable to other countries in similar settings.

On March 16, 2006, samples taken from a commercial turkey farm in southern Israel due to unexpected mortality rates (>0.7% per day) were positive for avian influenza subtype H5 by PCR. Highly pathogenic avian influenza (HPAI) subtype H5N1 was later confirmed by virus isolation. Eight more outbreak foci in commercial poultry farms in small settlements were identified within 2 weeks (Table 1). We briefly describe key findings of the outbreak investigation and lessons learned from our outbreak mitigation experience.

**Table 1:** Confirmed highly pathogenic avian influenza (H5N1) outbreaks in Israel*

Focus ID	District	Poultry type	Biosecurity standards	Date (2006)				Epidemiologic links (identifiers)			
				Increased no. deaths	Report	Diagnosis		Culling	FS	SH	Vet.
A	Southern	Meat type turkeys	Normal	Mar 14	Mar 15	Mar 16		Mar 17	A	A,B	A
B	Southern	Meat type turkeys	Normal	Mar 14	Mar 16	Mar 16		Mar 18	B	A	B
C	Southern	Meat type turkeys	Normal	Mar 15	Mar 16	Mar 17		Mar 20	A	B	A
D	Jerusalem	Meat type turkeys	Normal	Mar 15	Mar 16	Mar 17		Mar 18	A	A	D
E	Southern	Broilers	Normal	Mar 13	Mar 19	Mar 19		Mar 21	A,C	C	E
F	Southern	Heavy breeders	High	Mar 19	Mar 19	Mar 19		Mar 21	D	*	F
G	Jehuda and Samaria	Meat type turkeys	Normal	Mar 21	Mar 21	Mar 22		Mar 23	D	*	G
H	Jerusalem	Heavy breeders	High	Mar 28	Mar 28	Mar 28		Mar 30	A	*	E
I	Southern	Meat type turkeys	Normal	Mar 30	Mar 31	Mar 31		Apr 1	A	*	B

*No slaughtering took place in the 30 days before or during the outbreak period. ID, identifier; FS, feed supplier; SH, slaughterhouse; Vet., veterinarian.

## The Study

Epidemiologic investigation was performed by a joint team of veterinary and public health epidemiologists, administrators, and law enforcement officials. Descriptive epidemiology as well as cross-matching of available records were also performed to identify common factors associated with >2 foci and to establish potential flow of events. These records included log books of the affected farms, which contained daily mortality rates, identification of vehicles entering, personnel working and visiting the farm in the previous 30 days, and affiliated slaughterhouses.

In February 2006, influenza virus (H5N1) was detected for the first time in Egypt (*1*); in March 2006, outbreaks were detected simultaneously in the Palestinian Authority’s Gaza Strip and Israel. Later in March 2006, a single case was detected in Jordan (*2*). Molecular characterization of the isolates from Israel and Gaza performed in the Veterinary Services Central Laboratory showed that they were different from influenza (H5N1) viruses recently isolated in Indonesia (*3*); they belonged to a single strain and were closely related to other HPAI (H5N1) strains isolated during this period in European, Asian, and African countries.

Turkey farms, accounting for 10% of Israeli poultry farms, were unproportionally involved in this outbreak (6/9 outbreak foci). The relative prevalence of turkey farms in the southern district near the Gaza Strip (50% of farms); the close interactions between personnel at farms of the same poultry type; and the higher susceptibility of turkeys to avian influenza virus (*4*) may be plausible explanations.

Several epidemiologic links between outbreak foci were identified (Table 1). These links and the near-simultaneous detection of several outbreak foci specifically on turkey farms, increase the likelihood that the virus disseminated through use of shared vehicles or by personnel. Alternatively, the involvement of 2 heavy breeder farms (farms F, H) characterized by strict biosafety procedures to prevent such transmission, and the fact that all 9 farms used open sheds, may support the role of migratory birds in disease transmission.

Because all epidemiology-trained veterinarians were assigned to regional outbreak containment at multiple foci, initiation of coordinated epidemiologic investigation in the farms was delayed by up to 10 days. Therefore, precrisis allocation of designated epidemiology-trained veterinarian investigators and joint investigation team training could be an important component of avian influenza preparedness plans.

The key control measures taken, the case definitions used, and the guiding principles for oseltamivir prophylactic treatment are summarized in Table 2. Israeli-Palestinian cooperation allowed coordination of cross-border mitigation efforts (*5*). Overall, these control measures enabled full outbreak containment within 17 days, without further recurrences (as of August 2007).

**Table 2:** Veterinary and public health measures taken during the highly pathogenic avian influenza (H5N1) outbreak in Israel, by proximity to infected poultry

Measure taken	Location by proximity to outbreak focus			
	Infected flock	Protection zone (<3 km)	Surveillance zone (3–10 km)	Outside outbreak area (>10 km)
Management of poultry	Stamping out	Stamping out	Active surveillance: transportation of poultry and hatching eggs allowed only following PCR testing of samples within the previous 72 h	Passive and active surveillance
Poultry products management	Destroyed	Destroyed	Released for consumption after clinical examination of the laying flocks proved negative	No restrictions
Poultry contacts monitoring	Self-monitoring	Self-monitoring	None	None
Case definition of human suspected avian influenza	Close contact with poultry and any ILI*	Close contact with poultry and severe ILI†	None	None
Oseltamivir prophylaxis to poultry contacts	All poultry contacts (including all culling and burial teams)	All poultry contacts (including all culling and burial teams)	None	None

*ILI, influenza-like illness: respiratory symptoms and fever (>37.5^o^C). †ILI as defined above, in severity that requires hospitalization.

Rapid recruitment of teams willing and able to take part in culling and burial proved highly challenging. The Israeli Ministry of Defense was therefore assigned to coordinate and execute these efforts (through its civilian contractors) and did so effectively. Teams involved in poultry eradication activities were instructed to use N95 masks, disposable gowns, and safety goggles. Yet in hindsight, the investigation showed that, in some cases, the equipment was not used properly (e.g., gowns left open, mask lowered to uncover the nose) due to the challenging physical conditions in the hot and humid poultry houses. Shorter work shifts within the farms and better education of uninitiated workers are therefore key logistical aspects of preparedness.

Oseltamivir chemoprophylaxis (75 mg/day until 7 days after last exposure to poultry) was given to all culling teams, including poultry workers in the 3-km protection zones surrounding the infected farm. This policy was in accordance with European Center for Disease Prevention and Control guidelines (*6*), but not with the guidelines of Centers for Disease Control and Prevention (*7*) or the World Health Organization (*8*) that recommend against providing prophylactic treatment to low-risk exposure groups. This extensive approach proved helpful in recruiting culling workers, relieving their fears, and in reassuring the local population potentially exposed to the infected poultry. Also, only a minute fraction (425 prophylactic courses) of the Israeli pandemic preparedness stockpile had to be used.

Timely and full (market price) compensation to farmers was key in encouraging prompt reporting and achieving trust and cooperation of poultry owners in culling and gathering epidemiologic data. Culling was performed by administering organophosphate poison in the flock’s drinking water after 24 hours of water deprivation. This method proved lacking, as not all birds died as a result of this process. In certain cases, birds had to be manually slaughtered, a method that potentially exposes workers to increased risk for infection. Alternative culling methods such as use of asphyxiating foam are now considered for future outbreaks. The birds were buried above large polyethylene sheets within or in close proximity to the farm, and lime was applied to accelerate decomposition. Composting, a more environmentally friendly method that prevents ground water contamination (*9*), is considered for healthy birds culled in the protection zone. Only a few valuable birds (i.e., in zoos) were vaccinated with stockpiled H5N2 vaccines, because in some cases these vaccines may increase circulation of H5N1 viruses by allowing asymptomatic infections (*10**,**11*) potentially leading to continuous silent spread of the disease among birds (and subsequently to humans).

The Israeli public proved quite attentive to risk communication efforts as shown by the results of a national telephone survey conducted at the peak of the outbreak by the Israeli Center for Disease Control. Among a random sample of Israelis >21 years of age, 34 (62%) of 552 interviewees who were aware of the outbreak and generally consumed poultry products did not reduce poultry consumption at all due to the outbreak. In contrast, a recent preevent survey in the United States has shown that 40% of respondents would stop eating poultry products altogether if the H5N1 virus was detected. (*12*). This outbreak was also not associated with a massive increase in “worried well” hospital admissions. Only 24 patients (21 adults and 3 children) came to local hospitals due to self-defined or general practitioner-defined suspected avian flu during March 2006. Five of these 24 persons (4 adults and 1 child) indeed met the case definition of suspected case and were hospitalized, but none had laboratory-confirmed H5N1 infection. These results are probably derived, at least in part, from the effective frontline risk communication efforts of the district health officers who offered guidance to local general practitioners and the anxious public at the outbreak scene.

## Conclusions

Preparedness planning for avian influenza should account for the unique challenges associated with a simultaneous multifocal outbreak, including personnel recruitment and allocation; coordination of all parties involved in outbreak mitigation and investigation; simultaneous culling and disposal in multiple sites; and coordinated central and local risk communication efforts. Outbreak containment, even in these settings, could be achieved without the use of vaccines, which should be kept as a measure of last resort. Case definition and antiviral prophylactic policies may be revised ad hoc according to the unfolding events and in response to the medical and psychological needs of each population. The lessons learned and described in our study may serve to refine preparedness plans elsewhere in view of the increasing global dissemination of this virus.
